# Supplementary material for: The GluTR-binding protein is the heme-binding factor for feedback control of glutamyl-tRNA reductase
Source: eLife. 2019 Jun 13;8:e46300. doi: 10.7554/eLife.46300 (PMC6597238; doi:10.7554/eLife.46300)
Supplement: Supplementary file 1. [file elife-46300-supp1.docx]

| Table SII: Overview of primers used for qRT PCR analysis | | |
| --- | --- | --- |
| **name** | **description** | **sequence (5’-3’)** |
|  | **qRT PCR** |  |
|  |  |  |
|  |  |  |
| AtHEMA1 _fwd | Glutamyl-tRNA-reductase 1 | TTGCTGCCAACAAAGAAGAC |
| AtHEMA1 _rev |  | CCGTCTCCAATGAATCCCTC |
| AtHEMA2 _fwd | Glutamyl-tRNA-reductase 2 | AGAAGATTAGAGCAAAGGTGGA |
| AtHEMA2 _rev |  | TTCACCCTCTACTCAAGTGTG |
| AtGSA1 _fwd | Glutamat-1-semialdehyd-aminotransferase 1 | TCAAAGAAGAGCGACACAGAG |
| AtGSA1 _rev |  | GTAAACACCTTCTTCCAACATTCC |
| AtCHL27 _fwd | MgPMME cyclase subunit | GCTTCTTCTGCCTCTCGGTTTATG |
| AtCHL27 _rev |  | GCCGTGGTTCGGTTTGTCTCG |
| AtFC1_fwd | Ferrochelatase 1 | AGCCGTTCCAGTCAGTTTCGT |
| AtFC1_rev |  | CTCCATGTCTATCTCCTCAAGTGTCT |
| AtFC2_fwd | Ferrochelatase 2 | AGAGAATATTTCGAGAGGAC |
| AtFC2_rev | Scharfenberg et al. 2015 | CTCCATGATTAGATCAACAC |
|  |  |  |
